# Associations of early marriage and early childbearing with anemia among adolescent girls in Ethiopia: a multilevel analysis of nationwide survey

**DOI:** 10.1186/s13690-021-00610-7

**Published:** 2021-06-03

**Authors:** Fentanesh Nibret Tiruneh, Mesfin Wogayehu Tenagashaw, Degnet Teferi Asres, Hirut Assaye Cherie

**Affiliations:** Department of Applied Human Nutrition, Faculty of Chemical and Food Engineering, Bahir Dar Institute of Technology, Bahir Dar University, Bahir Dar, Ethiopia

**Keywords:** Anemia, Adolescents, Early marriage, Early childbearing, Community-level, Individual-level

## Abstract

**Background:**

Early marriage and early childbearing are common practices in Ethiopia. Girls who get married and give birth at a very young age are more likely to experience several health problems including anemia among others. However, the effects of early marriage and early childbearing on anemia status of adolescent girls have not been quantified in previous studies. In this study, we assessed whether early marriage and early childbearing measured at both individual and community levels are associated with adolescent anemia.

**Methods:**

We analyzed data from the 2016 demographic and health survey of Ethiopia. Our study focused on 3172 late female adolescents (15–19 years). We used the chi-squared test and spearman correlation coefficients for bivariate analysis. The relationship between early marriage and childbearing with anemia was evaluated using multilevel binary logistic regression models while controlling other determinants.

**Results:**

Overall prevalence of anemia among female adolescents was 23.8% (95% CI; 22.3–25.2). Our multivariable multilevel analysis showed that individual-level marital status (AOR = 1.53, 95% CI = 1.06–2.02) and community-level childbearing status (AOR = 2.80, 95% CI 1.25–6.29) were positively associated with anemia among female adolescents.

**Conclusion:**

Our findings show the presence of significant association between early marriage & early childbearing with adolescent anemia. Therefore, there is a need for effective policies and programs to end the practice of early child marriage and the consequent adolescent pregnancy in Ethiopia. This will help to improve nutritional status of adolescent girls as well as nutritional outcomes of their children.

## Background

Adolescence has been described as the period in life when an individual is no longer a child, but not yet an adult. WHO defines ‘Adolescents’ as individuals in the 10–19 years age group [1]. Furthermore, adolscents are classified into early (10–14 years) and late (15–19 years) age groups [2]. There are more than 1.1 billion adolescents worldwide and approximately 85% of them live in developing countries [3].

Adolescents are nutritionally vulnerable groups due to the rapid growth and development which occurs at this age. Being at their growth spurt, their dietary needs are high and this makes them to be susceptible to anemia and other micronutrient deficiencies [4]. The risk of anemia is greater in female adolescents than males [5]. Increased iron requirements, excessive menstrual losses and nutritional deprivation aggravate and worsen pre-existing anemia and its adverse effects among females [6]. Studies showed that compared to early teens, late female adolescents are more likely to be anemic [7].

Early marriage (marriage before the age of 18) is associated with many social, physical, and health problems and it is common in many developing countries [8]. Women who get married early tend to have less schooling; begin earlier childbearing and care, and have less household decision-making power on their health. In addition, domestic abuse is much more likely to happen to early married women [9]. Early marriage is common in Ethiopia with 40% of women age 20–24 first married or in a union before age of 18. Girls who get married at a very young age are more likely to experience several health problems including anemia, depression and anxiety [10, 11]. Moreover, women who are engaged in marriage at young ages have lower satisfaction with life, self-esteem, and perceptions of gender equality which further affects their health and health related behaviors [12].

Early pregnancy is a serious public health concern due to its potential impact on maternal and child health. Adolescent pregnancy raises the risk of several health complications such as anemia, hypertension, preterm labor, maternal mortality, perinatal and neonatal mortality and low birth weight [13, 14]. Childbearing during adolescence is also observed to inhibit young girls’ post-menarcheal linear and significant development during a potential window of opportunity for catch-up development in an undernourished population. In addition, pregnancy and lactation at early gynecological age contribute to the depletion of micronutrients like iron and increase the susceptibility of young mothers to anemia. When adolescents reach pregnancy with low nutritional reserves, this condition is exacerbated [15]. Furthermore, early marriage and early childbearing affect adolescent nutritional status by lowering their educational attainment and work status which leads to lower-income, low autonomy and high fertility which together affect nutritional purchasing power, nutritional intake and other outcomes [16, 17]. Therefore, preventing early marriage and early childbearing not only improves women’s and children’s health but also help to reduce poverty and improve a country’s socio-economic status [18].

The effects of early marriage and early childbearing on adolescent anemia have not been quantified in Ethiopia. Moreover, multilevel analysis was rarely used to investigate community-based clustering of anemia. Community constitutes a key determinant of socioeconomic disparities in health including anemia status as it shapes individual opportunities and exposes residents to multiple risks and resources over the life course. Our study aimed to analyze the association between early marriage and early childbearing at both individual and community-level with anemia among late female adolescents in Ethiopia. We hypothesized that those adolescents who got married and given birth at early age are at higher odds of having anemia than their counterparts.

## Methods

### Data source

**Fig. 1:**
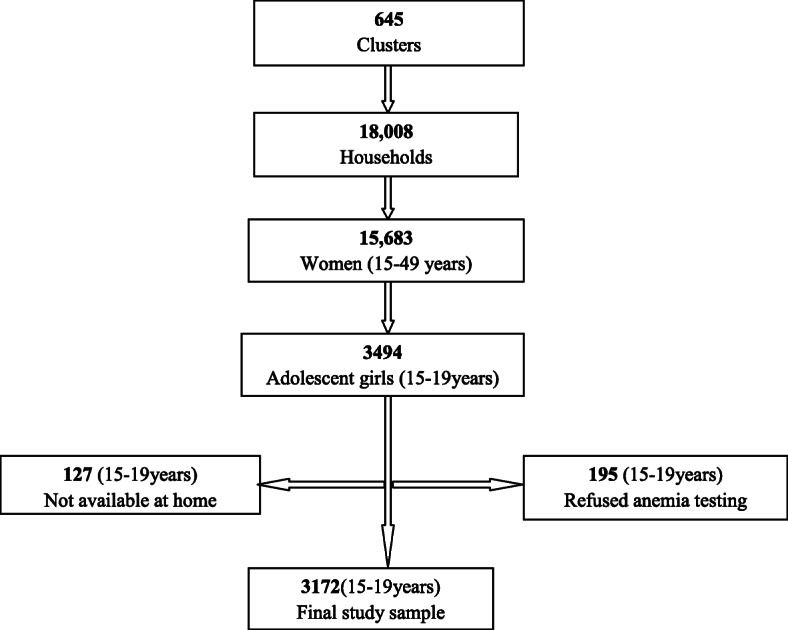
Flow diagram of the number of participants included in the study

We used data sourced from the 2016 Ethiopia Demographic and Health Survey (EDHS) for the analysis. The EDHS used a nationally representative sample and a stratified two-stage cluster sampling design. The sampling design involved randomly selected communities (clusters) at the first stage and households at the second stage. The sample comprised 645 communities (clusters) in the first stage and 18,008 households in the second stage. In total, 15,683 women age 15–49 years were interviewed. The overall response rate was 95%. Anemia testing was performed after getting consent from the participants. Among reproductive-age women, 3494 were adolescents (15-19 years). About 195 adolescents refused to be tested for their anemia status and 127 adolescents were not at home during blood collection. Therefore, our analysis was limited to 3172 female adolescents (15–19 years) making the response rate 90.7% (Fig. 1). Participants with hemoglobin levels below 7 g/dl (severe anemia) were referred for a follow-up care to a health facility. Besides, all households in which anemia testing was conducted were given a brochure explaining the causes and prevention mechanisms of anemia [19].

### Measurements

#### Outcome variable

Our outcome variable was anemia status among female adolescents which was caused by iron deficiency [19]. The cut-offs for anemia among women are defined by WHO as follows: mild anemia, when hemoglobin level is between 10.0 and 10.9 g per deciliter (g/dl); moderate anemia, when hemoglobin level is between7.0 to 9.9 g/dl and severe anemia, when hemoglobin level is less than 7.0 g/dl [20]. Due to very few numbers of cases in the category of severe anemia in the dataset, we have created two dichotomous variables where “0” denotes no anemia and “1” any anemia.

##### Individual-level independent variables

Individual-level variables included in our analysis were respondents’ marital status, religion, educational level, employment status, place of residence, nutritional status, pregnancy status, breastfeeding status, history of childbearing, use of contraceptives and visiting health facility in the last 12 months.

Wealth index was constructed using data on household’s ownership of selected assets such as television, materials used for constructing the house, etc. Factor scores of household assets were generated through principal component analysis and then standardized and categorized into quintiles (poorer, poor, middle, rich and richer).

##### Community-level independent variables

Based on the aggregation of individual responses for each item at the community (cluster) level, we assessed four community-level variables: proportions of female adolescents who got married, had secondary or higher-level education, had childbearing history and used contraceptives in the community. These variables represented the community norms and social contexts.

### Statistical analyses

All analyses were performed using SAS (version 9.4) software. We performed a series of bivariate analyses using chi-square tests to examine the association of individual- and community-level characteristics with the outcome variable (anemia). We also calculated Spearman correlation coefficients to analyze the relationship among community-level characteristics.

We further conducted multilevel multivariable binary regression models by using the “GLIMMIX” command in SAS to analyze the relationships between individual and community level explanatory variables with anemia.

Assuming that adolescents who are living in the same community to be more similar to each other than adolescents from different communities, the multilevel binary logistic regression models were used to adjust the correlated individual responses nested under the same community. We reported intraclass correlation coefficients (ICC) to assess the extent to which community variances were explained in each model.

### Model building

Four models were created. The first model (empty model) included the outcome variable only to check the variance in anemia between communities. The second model (model with only individual-level variables), the third model (model with only community-level variables) and the final multilevel model with both individual level and community level variables fitted simultaneously with anemia. The fixed effects for the multilevel binary logistic regression model were reported as adjusted odds ratios (AORs) with 95% confidence intervals (CI). The random effects were assessed using the intraclass correlation coefficient (ICC). Goodness of fit for each model was assessed using deviation information criterion, while the variance inflation factor and tolerance were used to examine multicollinearity. No multicollinearity problem was observed among the independent variables in the regression models (all tolerance > 0.1 and all VIF < 10).

### Ethics statement

The survey protocol, including biomarker collection, was reviewed and approved by the Federal Democratic Republic of Ethiopia Ministry of Innovation and Technology and the Institutional Review Board of ICF International. Informed consent was obtained at the beginning of data collection and consent for anemia testing was obtained from adolescents’ caretakers (parent/responsible adult). The authors sought consent from the EDHS program to use the data.

## Results

Table 1 shows individual and community-level characteristics of female adolescents participated in our study. About 727(23%) adolescents were married and 2067 (65%) of them live in rural areas. Regarding religion, 1310 (41.3%) of them were Orthodox Christians followed by Muslims 1255(39.5%). Around 331(10.4%) adolescents had early childbearing history; about 200 (6.3%) respondents used contraceptives and 1005 (31.7%) adolescents were underweight.

**Table 1:** Individual and community-level characteristics of female adolescents in Ethiopia (*n* = 3172)

	Frequency	%	Anemia	
			No	Yes
**Individual-level Characteristics(*n*** **= 3172)**				
Marital status				
Never married	2445	77.0	78.32	21.9**
Married	727	23.0	69.05	30.9
Religion				
Orthodox	1310	41.3	84.4	15.6**
Muslim	1255	39.5	66.9	33.1
Protestant	568	17.9	78.4	21.6
Others	39	1.2	69.2	30.8
Place of residence				
Urban	1105	34.8	81.4	18.6*
Rural	2067	65.2	73.4	26.6
Level of education				
No Education	504	15.9	60.91	39.1*
Primary education	1859	58.9	77.1	22.9
Secondary and higher education	809	25.5	83.6	16.4
Adolescent’s employment				
No	2389	75.3	75.0	25.0*
Yes	783	24.7	80.0	20.0
Wealth Index				
Poorer	715	22.5	61.7	38.3*
Poor	379	12.0	77.3	22.7
Middle	435	13.7	78.2	21.8
Rich	457	14.4	81.2	18.8
Richest	1186	37.4	82.0	18.0
Currently pregnant				
No	3065	96.6	76.7	23.3*
Yes	107	3.4	61.9	38.3
Currently breastfeeding				
No	2944	92.8	76.8	23.2*
Yes	228	7.2	68.4	31.6
Early childbearing				
No	2841	89.6	77.5	22.5**
Yes	331	10.4	65.3	34.7
Current use of contraceptive				
No	2972	93.7	75.8	24.2*
Yes	200	6.3	82.5	17.5
Nutritional status (BMI)				
< 18.5(underweight)	1005	31.7	71.1	28.9**
18.5–24.9 (normal weight)	2023	63.8	78.8	21.2
≥ 25 (overweight)	144	4.6	75.0	25.0
Visiting health facility in the last 12 months				
No	2338	73.7	76.3	23.7
Yes	834	26.3	75.9	24.1
**Community-level characteristics(*n*** **= 645)**				
% of married adolescents in the community				
Low	396	61.4	79.1	20.9**
High	249	38.6	71.9	28.1
% of adolescents who had childbearing history in the community				
Low	418	64.8	78.6	21.4**
High	227	35.3	72.5	27.5
% of adolescents with secondary and higher education in the community				
Low	286	44.3	73.2	26.8**
High	359	55.7	78.8	21.1
% of adolescents who use contraceptive in the community				
Low	489	75.8	73.5	26.5**
High	156	24.2	85.0	15
***Outcome variable (Anemia)***				
No anemia	2417	76.2		
Anemia	755	23.8 (95% CI; 22.3–25.2)		

**P < 0.001, p < 0.05; Data source EDHS, 2016

The prevalence of anemia among female adolescents was 23.8% (95% CI; 22.3–25.2). Our chi-square test showed that almost all variables had significant associations with anemia both at 0.01 and 0.05 significant levels.

Among communities, an average of 58.8% adolescents had secondary and higher education. Overall, 26.1% of the respondents were married; 11.2% had history of early childbearing and 8.4% used contraceptives (Table 2).

**Table 2:** Pearson correlation matrix among community-level characteristics (*n* = 645)

	Mean (standard deviation)	A	B	C	D
A. Percentage of adolescents with secondary and higher education	58.8 (30.9)	1.00			
B. Percentage of married adolescents	26.1 (25.4)	−0.02	1.00		
C. Percentage of adolescents with child bearing history	11.2 (10.9)	−0.04	0.41*	1.00	
D. Percentage of adolescents who used contraceptives	8.4 (9.7)	0.075	0.34*	0.19*	1.00

*P < 0.05

Table 3 depicts the results of our multilevel analysis. The empty model indicated that 29% of the variation of anemia among female adolescents was attributable to different factors that operate at the community level (ICC = 0.29; *P* < 0.001).

**Table 3:** Multilevel logistic regression

Variables	Empty model	Model with individual level variables AOR(95%CI)	Model with the community level variable AOR(95%CI)	Model with individual and community-level variables AOR(95%CI)
**Individual -level characteristics(*n*** **= 3172)**				
Marital status				
Never married		Ref.		Ref.
Married		1.54 (1.07–2.22)*		1.53 (1.06–2.20)*
Religion				
Orthodox		Ref.		Ref.
Muslim		2.85 (1.79–4.52)*		2.51 (1.56–4.01)*
Protestant		1.40 (0.84–2.31)		1.36 (0.81–2.25)
Others		7.68 (2.59–21.85)*		7.08 (0.88–19.02)
Place of residence				
Urban		Ref.		Ref.
Rural		0.86 (0.44–1.81)		1.03 (0.46–2.27)
Wealth Index				
Poorer		Ref.		Ref.
Poor		0.87 (0.64–1.19)		0.90 (0.66–1.22)
Middle		0.92 (0.66–1.27)		0.95 (0.69–1.32)
Rich		0.79 (0.56–1.12)		0.84 (0.59–1.18)
Richest		0.87 (0.57–0.98)*		0.91 (0.60–1.39)
Level of education				
No Education		Ref.		Ref.
Primary education		0.45 (0.34–0.65)*		0.48 (0.33–0.71)*
Secondary and higher education		0.21 (0.13–0.34)*		0.22 (0.13–0.35)*
Employment				
No		Ref.		Ref.
Yes		0.85 (0.72–0.99)*		0.85 (0.72–1.01)
Currently pregnant				
No		Ref.		Ref.
Yes		1.03 (0.76–1.40)		1.03 (0.76–1.40)
Currently breastfeeding				
No		Ref.		
Yes		0.89 (0.74–0.97)*		0.89 (0.74–1.08)
Early childbearing				
No		Ref.		Ref.
Yes		1.22 (0.77–1.95)		1.12 (0.70–1.80)
Current use of contraceptive				
No		Ref.		
Yes		0.47 (0.26–0.85)*		0.57 (0.32–1.04)
Nutritional status (BMI)				
< 18.5		Ref.		Ref.
18.5–24.9		0.56 (0.44–0.33)*		0.58 (0.45–0.75)*
≥ 25		0.38 (0.21–0.69)*		0.43 (0.32.0.54)*
Visiting health facility in the last 12 months				
No		Ref.		Ref.
Yes		1.15 (0.91–1.33)		1.10 (0.94–1.30)
**Community-level characteristics(n = 645)**				
Percentage of adolescents with secondary and higher education in the community				
Low			Ref.	Ref.
High			0.64 (0.33–1.23)	0.61 (0.31–1.19)
Percentage of married adolescents in the community				
Low			Ref.	Ref.
High			1.52 (0.68–3.38)	0.79 (0.35–1.77)
Percentage of adolescents who had childbearing history in the community				
Low			Ref.	Ref.
High			4.12 (1.83–5.24)*	2.8 (1.25–6.29)*
Percentage of adolescents who used contraceptives in the community				
Low			Ref.	Ref.
High			0.16 (0.07–0.34)*	0.31 (0.14–0.69)*
Measures of variation				
ICC	0.29*	0.23*	0.25*	0.21*
Model fit statistics				
DIC (−2log likelihood)	7054.6	6919.68	7004.63	6904.29

*AOR* Adjusted odds ratio, *CI* confidence interval, *DIC* deviation information criterion, *ICC* intraclass correlation coefficient, *AIC* Akaike information criterion. * Significant at *P*-value < 0.05

The analysis of the outcome variable with individual-level variables revealed that adolescents’ marital status, religion, household wealth index, educational level, employment status, breastfeeding status, contraceptive use and nutritional status were significantly associated with anemia. This model also showed that 23% of the variation of anemia among female adolescents was due to differences at the community level (ICC = 0.23; *P* < 0.05).

In the final model, we put both individual and community-level variables simultaneously and examined their association with anemia. The results showed that married adolescents were 1.53 times (AOR = 1.53; 95% CI: 1.06–2.20) more likely to be anemic compared to non-married ones. Muslim adolescents were 2.51 times (AOR = 2.51; 95% CI: 1.56–4.01) more likely to develop anemia than Orthodox Christians. Adolescents who had primary as well as secondary & higher education levels had 0.48 and 0.22 times (AOR = 0.48; 95% CI: 0.33–0.71 and AOR = 0.22; 95% CI: 0.13–0.35 respectively,) lower odds of being anemic compared to adolescents who had no formal education. Adolescents who had normal and overweight nutritional status were 0.58 and 0.43 times (AOR = 0.58; 95% CI: 0.45–0.75 and AOR = 0.43; 95% CI: 0.32–0.54) less likely to be anemic than underweight adolescents. Moreover, female adolescents from communities with a higher proportion of childbearing adolescents were 2.8 times (AOR = 2.80; 95% CI: 1.25–6.29) more likely to develop anemia than adolescents from communities with a lower proportion of childbearing adolescents. On the other hand, adolescents from communities with higher proportion of contraceptive users had 0. 69 times (AOR = 0.31; 95% CI: 0.14–0.69) lower odds of having anemia compared to their counterparts. The ICC in the final model suggests that about 21% variation in anemia may be attributable to other unobserved community-level factors (ICC = 0.21; *P* < 0.05).

## Discussion

The overall prevalence of anemia among female adolescents (15–19 years) was 23.8%. Adolescent anemia is thus a moderate public health problem in Ethiopia [21]. Our finding is comparable to previous studies conducted in Southern and Northwest Ethiopia [22, 23]. However, the prevalence is comparatively lower than studies done in different parts of Ethiopia such as Ambo Town, West Shewa which reported a prevalence as high as 39% [24]; 29% prevalence among school adolescents from Haramaya, Eastern Ethiopia [25] and 27% from southwest rural Ethiopia [26]. The potential reason for this discrepancy could be the target population, geographical area and sample size variations. For instance, the current study was nationwide and included both in school and out of school adolescents, and considered rural, peri- urban and urban areas. Furthermore, there can be a number of reasons to explain the difference in the prevalence of anemia in different parts of Ethiopia. One plausible reason could be difference in the culture and food practice among the different regions of Ethiopia. Variations might be also related to regional differences (9.6–58.9%) in early marriage and early child bearing practices [27]. Early marriage/child bearing can contribute to poorer health outcomes [28]. This is also noted in our study as married adolescents were more likely to be anemic than never married counterparts. A similar finding was reported from India as well [29]. The possible explanation for this could be early married adolescents are vulnerable to pregnancy and birth-related bleedings as well as other complications that may raise the risk of anemia [30]. Early marriage and early childbearing also affect adolescent nutritional status indirectly. First, as marriage is often cited as an important factor in dropping out of school and college among adolescents in developing countries including Ethiopia, it has a negative impact on their educational achievement [16]. In addition to this, low levels of education have an effect on employment status and contribute to lower incomes, low autonomy and high fertility which together affect nutritional purchasing power, nutritional intake behavior and outcomes [17]. Anemia during adolescence has adverse effects on growth, cognitive development, physical capacity and work performance [20]. Furthermore, adolescent anemia has been reported to increase risk of psychiatric disorders, including mood disorders, autism spectrum disorder, attention deficit hyperactivity disorder, and developmental disorders [31]. Iron-folic acid supplementation, food fortification and dietary diversification are some of the strategies mentioned in the national nutrition program [32] to prevent iron deficiency anemia among women of reproductive age group in Ethiopia. Iron-folic acid tablets are usually given for pregnant women during their antenatal care visits. However, compliance is generally low with great regional variations, 3.5% [33] to 76% [34] and some of the strategies proposed such as food fortification have not yet implemented.

Muslim adolescents had higher odds of developing anemia compared to Orthodox Christians. The potential explanation for this finding may be due to religious adherents. The norms and principles of the Muslim religion encourage large family size, early marriage and lesser use of contraceptives compared to Orthodox Christians [29, 30]. In 2013, the government of Ethiopia announced its commitment to end early or child marriage by 2025 in the National Strategy and Action Plan on Harmful Traditional Practices against Women [35]. Article 648 of the Criminal Code also makes child marriage illegal with a punishment of those involved in the arrangement of the marriage from 3 to 7 years [36]. Despite this policy or legal environment, early marriage is common in Ethiopia being seen by many as the best way to preserve girls’ (as well as their families’) honor and place in the community [36].

Our study revealed that adolescents’ educational level was inversely associated with anemia. Adolescents who had some kind of formal education had lower odds of having anemia than adolescents who had no formal education. The possible explanation for this finding could be related to early marriage and childbearing as adolescents with at least some secondary schooling are less likely to get married at a very young age than uneducated ones. Furthermore, education is positively associated with contraceptive use which can prevent early childbearing and reducing the risk of anemia [37].

Adolescents who had normal and overweight nutritional status were less likely to be anemic compared to underweight adolescents. Our result was consistent with reports of previous studies [38, 39]. Undernourished adolescents have more chances to be deficient in one or more micronutrients which may result in an increased risk of anemia [40]. In light of our results, we recommend that national anemia prevention programs should work on promoting safe dietary practices to maintain a healthy BMI and promote iron supplements to ensure optimal nutrition.

The effect of community characteristics on individual health outcomes and behaviors has been reported by prior studies [41, 42]. Our analysis revealed that unlike individual-level childbearing status, community-level adolescent’s childbearing was positively associated with anemia. Our hypothesis is partially supported by this finding. Even after we adjusted for childbearing status and other individual and group-level characteristics, an adolescent living in a community with a higher percentage of adolescents who had childbearing stories was more likely to have anemia. The potential reason may be related to socio-cultural norms as adolescent births are irrefutably related to the cultural and social norms of a society [43]. Some social norms such as child / early marriage and early childbearing may adversely affect adolescent health including being anemic. In most African countries including Ethiopia, married adolescents are expected to become pregnant within a year after marriage. As a consequence, Ethiopia has one of the highest teenage births with around 143 per 1000 girls between the ages of 15–19 falling pregnant [44]. Restricted social and decision-making autonomy for female adolescents also impacts health preferences, including the use of contraception for early married adolescents to prevent early childbearing [45]. Similar findings were also reported from some regions of India [46] and Brazil [47] where practices of early child marriage and early child bearing are common. Occurrence of pregnancy during adolescence with anemia increases not only maternal morbidity & mortality but also the incidence of poor maternal birth outcomes such as still birth, low birth weight, prematurity as well as low infant iron status [13, 48].

This study revealed that in addition to community-level adolescents’ childbearing status community-level contraceptive use was also associated with adolescents’ anemic status. An adolescent living in a community with a higher proportion of contraceptive users had lower odds of having anemia. This could be justified by adolescents who used contraceptives prevent complications related to early pregnancy and childbirth, which could eventually reduce the prevalence of anemia due to recurrent blood loss. Another plausible justification might be the use of contraceptives could minimize menstrual bleeding and reduce their susceptibility to anemia [49, 50]. Furthermore, adolescents who use contraceptives are more likely to be educated and autonomous and will subsequently be aware of their health and health related behavior. A higher concentration of autonomous adolescents in the community can promote the dissemination of information via informal social networks to those with lower autonomous peers [51].

### Study limitations

There are some possible limitations to this study. First, the use of data obtained from a cross-sectional study makes it difficult to infer causality between independent variables (early marriage and childbirth) and the outcome variable (anemia). Besides, adopting community-based measures by aggregating individual responses to the community level may have increased the probability of misclassification of individuals to inappropriate administratively defined boundaries (clusters) potentially leading to information bias. Despite these limitations, our study used a national representative large sample and therefore, results from the current analysis may be generalized to Ethiopian female adolescents (15-19 years).

## Conclusion

Our study demonstrates the importance of considering both individual-and community-level characteristics when investigating the effects of early marriage and childbearing on adolescent anemia. This study also revealed the presence of significant association between early marriage & early childbearing with adolescent anemia. Therefore, there is a need for effective policies and programs to end the practice of early child marriage and the consequent adolescent pregnancy in Ethiopia. This will help to improve nutritional status of adolescent girls and nutritional outcomes of their children.

## Data Availability

Dataset analyzed in the current study is available in https://dhsprogram.com/data/dataset/Ethiopia_Standard-DHS_2016.cfm?flag

## Abbreviations

AOR: Adjusted Odd Ratio
BMI: Body mass index
CI: Confidence Interval
DIC: Deviation Information Criterion
EDHS: Ethiopia Demographic Health Survey
ICC: Intraclass Correlation Coefficient
SAS: Statistical Analysis Software
VIF: variance inflation factor
WHO: World Health Organization
